# Vegan Glucosamine versus Crustacean Glucosamine in Osteoarthritis: Choosing the Correct One During Clinical Practice

**DOI:** 10.31138/mjr.201223.vgv

**Published:** 2024-04-23

**Authors:** Hitesh Verma

**Affiliations:** Biofern Life Sciences Pvt Ltd, Vemgal Industrial Area, Kolar District, Karnataka, India

**Keywords:** glucosamine, crustacean glucosamine, shrimp glucosamine, vegan glucosamine, clinical practice

## Abstract

The present commentary critically evaluates the role of glucosamine in joint health, specifically exploring the potential of vegan glucosamine as an alternative of crustacean glucosamine. While acknowledging glucosamine’s established benefits in managing osteoarthritis, this commentary underscores concern regarding the limited data supporting the clinical use of vegan glucosamine. Methodological flaws in the bioequivalence study, lax quality parameters, and the absence of safety data for E. coli-derived vegan glucosamine are highlighted. The commentary calls for caution in endorsing vegan glucosamine for osteoarthritis patients, stressing the need for further research and a thorough evaluation of its efficacy and safety before widespread clinical adoption.

Glucosamine, a naturally occurring amino sugar, serves as a foundational building block for various molecules in the body, especially those present in connective tissues like cartilage. It is chemically derived from glucose, a basic sugar, and falls under the classification of an amino monosaccharide. Glucosamine plays a crucial role in synthesising glycosaminoglycans and proteoglycans, which are essential components of cartilage and other connective tissues. In the realm of joint health, glucosamine is acknowledged for its contributions to maintaining and repairing cartilage. As a pivotal element of glycosaminoglycans, glucosamine reinforces the structural integrity of cartilage, ensuring its elasticity and shock-absorbing properties. Although the human body can internally produce glucosamine, its availability may decline with age or in specific health conditions. Consequently, glucosamine is frequently obtained through dietary sources or as a supplement to support joint health, especially in individuals dealing with conditions such as osteoarthritis (OA). Considerable research has been conducted to assess the efficacy of glucosamine supplementation in managing OA.^1,2^ A recent meta-analysis,^3^ which included data from 3949 participants, confirmed that glucosamine, when administered at a dosage of 1500 mg/day, can positively impact cartilage structure, alleviate pain, enhance functionality, and improve glucose metabolism in people with knee OA. Importantly, this positive effect occurs without a higher incidence of adverse effects compared to a placebo.

A diverse range of glucosamine salts are available in the market, encompassing glucosamine sulphate potassium chloride, glucosamine sulphate sodium chloride, glucosamine hydrochloride, glucosamine derived from fungus, and vegan glucosamine obtained from corn through fermentation (vegan glucosamine).^4^ While the first three salts have undergone extensive research and are backed by over decades of clinical practice, newly developed glucosamine salts, like vegan glucosamine, lack concrete data. The purpose of this commentary is to underscore various considerations that should be carefully weighed before integrating vegan glucosamine into clinical practice.

In the limited research available on the impact of vegan glucosamine on gut health^5^ and its bio equivalency with respect to marine glucosamine,^6^ it has been suggested that vegan glucosamine may offer advantages over crustacean-derived glucosamine for several reasons. Firstly, it is considered a sustainable source of glucosamine. Secondly, there is a reduced risk of allergic reactions compared to glucosamine derived from marine sources. Thirdly, it is deemed environmentally friendly.^6^ A comprehensive evaluation of these assertions, coupled with a critical analysis of its bioequivalence study when compared to shrimp-derived glucosamine, will be instrumental in making informed decisions in clinical practice. When considering the sustainability and environmental friendliness of crustacean-derived glucosamine, there is an ample supply of raw materials available for its production. Reports indicate that 1010 to 1011 tons of chitin are produced annually worldwide, with 2.5 million tons generated in Asian countries and approximately 1 million tons of shrimp waste produced each year.^7^ This approach is seen as a method to utilise biowaste, which is rich in nitrogen but can potentially pose serious environmental issues, such as ammonia toxicity if not properly managed.^7^ Contrary to concerns about sustainability, it appears that there is no immediate issue regarding the availability of raw materials for marine glucosamine. Furthermore, shellfish allergy is typically caused by IgE antibodies to antigens in the flesh of the shellfish and not the shell itself (from where glucosamine is produced after decalcification)^8^ As a result, it is generally considered safe for individuals with shellfish allergies to take glucosamine supplements. Clinical studies, specifically focusing on the usage of shrimp-derived glucosamine in patients with shellfish allergies, have been conducted and have concluded that glucosamine usage is safe and does not pose any significant risk of allergic reactions.^8,9^

Moreover, the bioequivalence study^6^ conducted to establish the equivalency of vegan glucosamine with marine glucosamine exhibits multiple methodological flaws. Notably, the sample size was small (n=10), and despite employing a crossover design, there is significant variability in the reported data. Additionally, the choice of a 90% confidence interval (CI) instead of the conventional 95% CI may lead to an overstatement of findings. Even within the 90% CI, the research fails to demonstrate equivalency across all pharmacokinetic parameters (such as T_max_ and terminal half-life), contributing to the observed high variability among subjects. Furthermore, comparing certificate of analysis of leading products used in bioequivalence studies and samples tested in our laboratory showed that vegan glucosamine is less stringent in quality parameters related to microbial contamination^10,11^ and residue left on ignition observed in our laboratory (0.01±0.002% for crustacean glucosamine versus 0.1± 0.04% in vegan glucosamine, *p*<0.05 using paired Student’s t-test). Also, there is a notable absence of toxicity and safety data for vegan glucosamine, while an ample history of clinical usage is available for crustacean-derived glucosamine. Vegan glucosamine is produced using engineered strains of *Escherichia coli,*^7^ and there is a lack of safety and toxicity reports specifically for *E. coli*-derived glucosamine. Although published research on vegan glucosamine does cite a reference for safety, the mentioned reference pertains to *Aspergillus niger* (a fungus)-derived glucosamine12 and lacks any reference to *E. coli*-derived glucosamine.

Therefore, despite the initial appeal of vegan glucosamine, there is in-sufficient data to support its clinical usage. This is primarily due to the absence of direct clinical studies of usage of vegan glucosamine in OA patients, the shortcomings in its bioequivalence studies, and the lack of its safety and toxicity data. Clinicians should carefully weigh these considerations before making clinical decisions.

## References

[1] Kirkham S Samarasinghe R. Review Article: Glucosamine. J Orthop Surg 2009;17(1):72–6. 10.1177/230949900901700116 19398798

[2] Vo NX Le NNH Chu TDP Pham HL Dinh KXA Che UTT Effectiveness and Safety of Glucosamine in Osteoarthritis: A Systematic Review. Pharmacy (Basel) 2023;11(4):117. 37489348 10.3390/pharmacy11040117 PMC10366893

[3] Veronese N Demurtas J Smith L Reginster JY Bruyère O Beaudart C on behalf on the European Geriatric Medicine Society Special Interest Groups in Systematic Reviews and Meta-Analyses and Arthritis. Glucosamine sulphate: an umbrella review of health outcomes. Ther Adv Musculoskelet Dis 2020;12(Jan–Dec):1–12. 10.1177/1759720X20975927 PMC7768322 33488785

[4] Ma Q Gao X. Categories and biomanufacturing methods of glucosamine. Appl Microbiol Biotechnol 2019;103:7883–9. 31440792 10.1007/s00253-019-10084-x

[5] Moon JM Finnegan P Stecker RA Lee H Ratliff KM Jäger R Impact of Glucosamine Supplementation on Gut Health. Nutrients 2021;13(7):2180. 34202877 10.3390/nu13072180 PMC8308242

[6] Kang HE Kim SJ Yeo E Hong J Rajgopal A Hu C Pharmacokinetic Comparison of Chitosan-Derived and Biofermentation-Derived Glucosamine in Nutritional Supplement for Bone Health. Nutrients 2022;14(15):3213. 35956389 10.3390/nu14153213 PMC9370395

[7] Suryawanshi N Eswari JS. Shrimp shell waste as a potential raw material for biorefinery—a revisit. Biomass Conv Bioref 2022;12(May 2022):1977–84.

[8] Gray HC Hutcheson PS Slavin RG. Is glucosamine safe in patients with seafood allergy? J Allergy Clin Immunol 2004;114(2):459–60. 15341031 10.1016/j.jaci.2004.05.050

[9] Villacis J Rice TR Bucci LR El-Dahr JM Wild L Demerell D Do shrimp-allergic individuals tolerate shrimp-derived glucosamine? Clin Exp Allergy 2006; 36(11):1457–61. 17083356 10.1111/j.1365-2222.2006.02590.x

[10] GlucosaGreen, D-Glucosamine HCl, Certificate of analysis, Item no GLH503, Batch no GLH20180403, 2018.

[11] Glucosamine Hydrochloride USP -43, Certificate of analysis, Item no GLH01, CAS no 66-84-2, Batch no GLHS11230042, 2023.

[12] Scientific Opinion of the Panel on Dietetic Products Nutrition and Allergies on a request from the European Commission on the safety of glucosamine hydrochloride from Aspergillus niger as food ingredient. The EFSA Journal 2009;7(6):1–19.

